# On the Prediction of Flickr Image Popularity by Analyzing Heterogeneous Social Sensory Data

**DOI:** 10.3390/s17030631

**Published:** 2017-03-19

**Authors:** Samah Aloufi, Shiai Zhu, Abdulmotaleb El Saddik

**Affiliations:** 1 Multimedia Computing Research Laboratory (MCRLab); School of Electrical Engineering and Computer Science, University of Ottawa, Ottawa, ON K1N 6N5, Canada; saloufi@mcrlab.uottawa.ca (S.A.); elsaddik@uottawa.ca (A.E.S.); 2 College of Computer Science and Engineering (CCSE), Taibah University, Medina 42353, Saudi Arabia

**Keywords:** social sensors, social sensory data, social image, popularity prediction, enhanced living environment, social media, social networks

## Abstract

The increase in the popularity of social media has shattered the gap between the physical and virtual worlds. The content generated by people or social sensors on social media provides information about users and their living surroundings, which allows us to access a user’s preferences, opinions, and interactions. This provides an opportunity for us to understand human behavior and enhance the services provided for both the real and virtual worlds. In this paper, we will focus on the popularity prediction of social images on Flickr, a popular social photo-sharing site, and promote the research on utilizing social sensory data in the context of assisting people to improve their life on the Web. Social data are different from the data collected from physical sensors; in the fact that they exhibit special characteristics that pose new challenges. In addition to their huge quantity, social data are noisy, unstructured, and heterogeneous. Moreover, they involve human semantics and contextual data that require analysis and interpretation based on human behavior. Accordingly, we address the problem of popularity prediction for an image by exploiting three main factors that are important for making an image popular. In particular, we investigate the impact of the image’s visual content, where the semantic and sentiment information extracted from the image show an impact on its popularity, as well as the textual information associated with the image, which has a fundamental role in boosting the visibility of the image in the keyword search results. Additionally, we explore social context, such as an image owner’s popularity and how it positively influences the image popularity. With a comprehensive study on the effect of the three aspects, we further propose to jointly consider the heterogeneous social sensory data. Experimental results obtained from real-world data demonstrate that the three factors utilized complement each other in obtaining promising results in the prediction of image popularity on social photo-sharing site.

## 1. Introduction

With the proliferation of social media and the pervasiveness of sensor-embedded portable devices, the boundaries between real life and the virtual world have vanished. Most of these devices are connected to the Internet and are capable of capturing and delivering various types of media that report on a wide range of topics. Every day, hundreds of millions of registered users are generating and sharing massive amounts of data on social networks and interacting with each other regardless of time and location. The shared data describe people’s interests, daily activities, opinions, and what is happening in their surrounding environment. Consequently, users on social media are acting as social sensors, providing large-scale data about their social and living environments that would be impossible to obtain from other sources [1,2]. Krishnamurthy and Poor in [3] defined a social sensor as "an agent that provides information on social networks about their environment after interaction with others". Social sensory data that are available on social media impact the decisions and choices of individuals and communities. Opinions and reviews of restaurants or hotels on TripAdvisor, Yelp, and Foursquare influence the choices of other people. On Twitter and Facebook, people post short messages about their feelings, opinions, and social events. Due to the real-time nature of posts on some social media platforms, people and authorities can find a large number of texts, photos, videos, or a combination of these media reporting real-world events and news. These events are not limited to social events, such as concerts, sports, and elections; they can also include information on emergencies and disasters such as earthquakes, storms, and traffic jams [4]. These examples demonstrate the merge that is occurring between the real world and social media. Notably, people are moving away from storing their photos and memories in physical albums and are now using social photo-sharing sites, such as Flickr and Instagram, where they become involved with a larger community. These social media services offer users features to store and organize their media, share it with other users, and document their memories via text-free descriptions and annotations. The mining of social sensory data contributes to the analysis of people’s opinions and behaviors and, in turn, can provide better services to the users and their environment through informed decision making, crisis management, product recommendations, and future trend predictions. Social data, however, are difficult to measure and process with physical sensors [3]. Although users’ opinions about products and their movie preferences are readily available, they cannot be measured using physical or hardware sensors. Moreover, the popularity of a product, an image or a video cannot be detected or predicted by traditional sensors.

Evidently, social data have created opportunities to understand the behavior of users and provide better services, yet they also present new challenges, especially from the data analysis perspective. Social data have unique characteristics that differentiate them from traditional data. This type of data is defined as huge amounts of diverse and noisy data with variations in quality, posted on social networks by users with different backgrounds combined with various types of metadata [5,6]. Additionally, social media data are interconnected, which is highly correlated with the social behavior that captures the perspective of communities or individuals in regards to the shared media [5]. This social behavior can be expressed explicitly by either liking, commenting or marking as a favorite or implicitly by simply viewing the item without taking any explicit action. Sharing content and interacting with others on social media is motivated by social presence and popularity [7]. Thus, users maintain a list of contacts or friends and subscribe to multiple self-organized groups to connect with people who share their interests. In addition, users on social media utilize the features provided by the service providers, such as hashtags, and text-free annotations, to boost the visibility of their content to other users. These unique characteristics of social data influence the social interactions and data propagation, which results in a nonstandard pattern of information flow [3]. Thus, understanding the dynamic of social data flow [3] and user social behavior helps build systems that are able to analyze and interpret social data to provide services that enhance the user’s living environment explicitly or implicitly. As an illustration of enhanced living environment examples, data collected from social media increase awareness during crises such as floods and fires [8]. Updates on construction and traffic jams help users navigate a route and avoid congestion. This is an explicit impact of social media on the physical world. Implicitly, providing users with more accurate and satisfying results to their inquiries on search engines saves them time and effort. Moreover, social data contribute in building systems that detect objects and faces in images, which can be used in authentication and security systems. In addition, the availability of images with rich textual information and geo-location data on social media sites, such as Flickr, are used to recommend places and attractive destinations to travelers.

**Figure 1:**
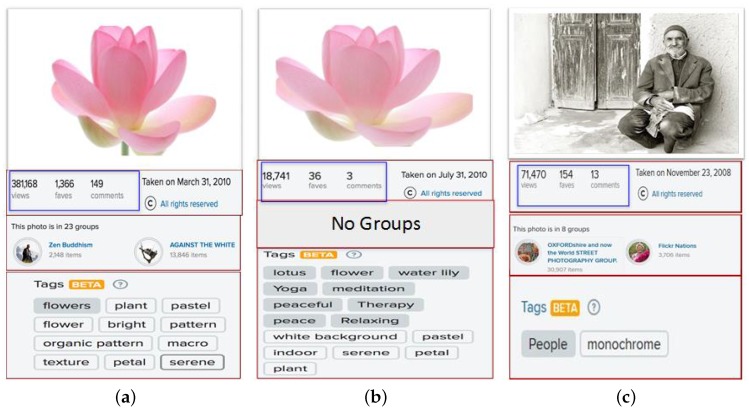
Variation in the number of social interactions between users and images. Visual content, social context, and textual information are important factors for making an image popular. In (**a**), and (**b**) the images share the same visual concept; in (**c**) the image represents different visual concept.

The above-mentioned examples demonstrate the importance of social interactions in social media data mining and analysis. In this paper, we take the opportunity to explore the factors that could impact the social interactions of social sensors “users” on social media which lead to content popularity. With the growth of social media, only a limited number of users and their content attract a great deal of attention from users; whereas, many others go unnoticed. When we consider images in social photo-sharing sites, we witness a variation in their number of views or “social popularity”, regardless of their visual appeal. This motivates us to answer the question: which factors impact the popularity of social content? Understanding the social behavior and the underlying structure of social photo-sharing sites will contribute to designing better prediction algorithms. We examine Flickr, a photo-sharing social network, in order to study the social interactions between users and images. Since Flickr has large amounts of publicly available photos and provides a comprehensive API, it allows us to collect information about images and users with their social context. Moreover, Flickr has various features that allow users to make their photographs accessible and visible to a large number of online users. In addition to sharing images with friends and group members, users on Flickr are encouraged to annotate their images with text-free tags so that they can be accessible via keyword searches. As depicted in Figure 1a–c are examples of images uploaded by the same user. In Figure 1a,b, the images share the same visual content; whereas in Figure 1c, the image represents a different visual concept. The three images in Figure 1 received different amounts of social interactions, even though the images had a similar visual appearance. This variation in the number of social interactions between the images is impacted by factors other than visual content. Figure 1 shows the differences between the images in terms of surrounding text represented by tags and the number of groups that an image is joining, which affect the number of social interactions. Attaching descriptive tags to the images and sharing them with suitable groups help in making them popular. Thus we study the relationship between social context, visual content, and textual information and social interactions. While the image’s visual semantic, sentiment and Aesthetic exhibit an impact on the social interactions, the user and the image social contexts play a significant role in the image’s popularity. Examples of such social context are the user’s contacts and the image’s groups. The effect of the textual information cannot be neglected when predicting an image’s popularity. Once the analysis is complete, we apply our findings to implement a social image popularity prediction algorithm. This work is an initial step that can guide users to boost their image’s popularity on photo-sharing social networks by showing the factors that will affect their social interactions as well as the topics that are more attractive to their contacts.

Prior studies attempted to address the topic of predicting an image’s popularity with content and/or social factors [9,10,11] by utilizing basic features. In [12], the authors combined social context with low level and deep learning features to determine what makes an image popular, while [13] only considered an image’s visual content using deep learning features to distinguish between popular and unpopular scenes that affect an image’s popularity. In [14], the authors addressed the visual sentiments that impact an image’s popularity. In our work, we address the problem by first scrutinizing the social behavior in Flickr to determine how users browse social photo-sharing sites and then analyzing the correlation between users and an image’s social factors with regard to social popularity. In addition, we investigate the role of associated text in an image’s popularity. We conclude that three factors, social behavior, visual content, and surrounding text, absolutely influence the photos visibility to other users and subsequently its popularity.

In this paper, we are targeting Flickr as our main platform in studying a social image popularity problem. First, we define the image popularity as the number of views or the number of comments and favorites. Then, we analyze the social and textual contexts that are available on Flickr and that may impact the image popularity in Section 3. Lastly, we propose a system to predict images popularity by implementing a prediction algorithm based on multi-modality features in Section 4. In the prediction system, we use content-based features, the social context of users and images, and textual features to predict the popularity ranking scores of a given set of images. From our data analysis, we determine that images may be posted in groups where the owners are not even members. These groups accept images by invitation and are considered prestigious groups in Flickr. Thus, we use image groups as one of the image’s social factors. Unlike prior works, we examine the effect of different levels of visual features that represent the semantic and sentiment concepts that appear in the image. Moreover, we consider investigating the influence of an image’s beauty on its popularity, especially when images are similar in visual content and social factors. Our experiments demonstrate the capability of visual, social, and textual features to predict an image’s popularity within different sets of images. In addition, we validate the benefit of combining multi-modal features in order to improve the performance of the prediction algorithm. Our prediction algorithm is designed for Flickr, and cannot be directly applied to other social networks due to the differences in available social and content features. Yet, the concept of utilizing multi-modal features that propose in our paradigm is applicable to other social networks.

We summarize the contributions of our work as follows:

- We provide a comprehensive study that analyze a real data collected from Flickr to explore the factors that could impact the social interactions. We have studied the relationship between the explicit and implicit social interactions and the social context of users and images.
- We further investigate the effect of visual content and surrounding text on the interaction analysis, which is defined as popularity prediction in this paper. Various visual features, including low level, middle level, and semantic features, as well as textual information are explored to provide a comprehensive understanding of this issue.
- Finally, we consider combining different studied factors and investigate their effect on predicting different levels of interactions. We demonstrate the impact on the algorithm’s performance as it integrates the three modalities of features. Our results show that when combining various types of features which represent different visual aspects, the performance of the algorithm is improved. For example, combining semantic level and low level features can improve the algorithm’s performance over using an individual feature. Moreover, features from different modalities provide enhancement on the prediction algorithm’s performance.

In the remainder of the paper, a review of related work is presented in Section 2. We then present the data collection and analysis in Section 3. In Section 4, we describe our approach in predicting image popularity. The experiments and result analysis are provided in Section 5. Finally, Section 6 includes a summary of our findings and a discussion of possible future work.

## 2. Related Work

An increasing number of studies propose to analyze social interactions in social media and utilize User-Generated Content (UGC) to address relevant multimedia and data mining problems. These studies cover a wide range of applications such as multimedia retrieval and re-ranking, personalized recommendations, image and video annotation, personality detection, inferring relationship strength, human behavior prediction and media popularity prediction. Our objective in this work is to understand the social interactions among different entities in social media that may reflect users’ living environments. Thus, we group previous work into two categories: (1) human social behavior on the Web; and (2) image interestingness and popularity.

### 2.1. Social Interactions between Users and Data

Much research has focused on analyzing social behaviors and information propagation in social media. In [15], Van Zwol studied the characteristics of users’ social behavior on Flickr. This work demonstrated that photos received the majority of their views within the first two days of being uploaded; therein, were influenced by the owners’ contacts and social groups to which he/she belonged. In addition, Van Zwol concluded that images with a high number of views are explored by users worldwide. Cha et al. [16] analyzed the propagation of photos on Flickr and observed a significant impact of a user’s social links on image popularity and propagation. Unlike [15], they found that the favorite marks that images received were coming from users within a few hops of the owner or network. This could be because they considered the number of favorites; whereas, the study conducted by Van Zwol [15] considered the number of views, where viewers are not required to be Flickr members and the favorite action is usually influenced by social relations. A recent study conducted by Lipczak et al. [17] analyzed the social behavior of users who mark photos as favorites on Flickr. They found that 50% of the favorite actions occurred within one week of an image’s upload date, and the largest portion of these favorites was from the owner’s contacts. Alves et al., in [18], studied the impact of users’ networks on favorite actions on Flickr. They reported that 70% of the favorite marks of photos were received from the owner’s contacts. Lerman and Jones [19] analyzed social behaviors on Flickr using sample images from the Explore page of Flickr, Apex group, which is one of the popular groups, and a random set of images. They analyzed the visibility of the images to users by investigating the size of a user’s network, the number of groups to which they belong, and the tags attached to the images. Their analysis showed a high correlation between an image’s popularity signals “views, favorites, comments” and the number of reverse contacts of the owners while tags were deemed less important. Social groups only correlated with random images’ popularity and showed no important role in Apex and Explore images. In another work, Cha et al. [20] analyzed the favorite behavior on Flickr and identified two patterns. The number of favorites for an image usually increases steadily, but certain images, at times, experience rapid growth because of external exposure. On the other hand, Valafar et al. [21] studied the favorite behavior on Flickr and reported that 10% of users are the cause of 80% to 90% of the favorite action. Additionally, they confirmed the findings of other studies asserting that photos will be discovered within the first week after being posted, after which the popularity will increase steadily during the photo’s lifetime. We can see that analyzing human social behavior is a challenging task; thus, a stable and unified pattern is difficult to model. This is in contrast to other media types or platforms. For instance, Youtube videos are constantly viewed by users throughout their time online; whereas, news often reaches a saturation point within a few hours of being posted [22].

### 2.2. Image Interestingness and Popularity

Defining image interestingness is difficult because this subjective concept is based on a user’s preferences. Despite the difficulty, we can still observe agreement among a large number of online users on image interestingness, especially with the proliferation of social media. This phenomenon attracts researchers from computer vision and multimedia domains who attempt to identify the reasons that certain photos are considered popular or interesting. The studies conducted either utilize a single modal or a multi-modal approach that combines multiple features that are available in social networks and the content itself to predict an image’s popularity. Because users on social media sites can view, comment and select as a favorite any image to express their interest, these social metrics can be utilized to indicate a photo’s popularity [16]. In addition to the utilization of different social metrics to predict popularity, researchers have modeled the problem of popularity prediction with several learning paradigms.

Van Zwol et al. [23] modeled the prediction of whether a given user will select an image as a favorite as a binary classification using Gradient-Boosted Decision Trees (GBDT). The learning algorithm was trained based on visual, social and tag features. They reported that, in most cases, combining social and visual features achieved the best results in predicting the image that a user will mark as his/her favorite. McParlane et al. [11] also addressed popularity prediction as a binary classification by training a non-linear SVM classifier. They considered the cold start scenario, where interaction information is not available. In the cold start case, there is limited textual and interaction information; therefore, information related to image and user contexts was utilized. They used the number of views and comments as the two social popularity metrics to classify images into low- or high-popularity classes. Each image was represented as a binary vector according to the extracted visual feature, social clues and textual information. The results showed that a user’s social context played a significant role in predicting image popularity.

Meanwhile, Hsieh et al. [24] found a weak correlation between an image’s Aesthetics and its popularity on social media. Thus, they attempted to determine which image features led to social popularity and concluded that, among basic image features, color was the most important. This work verified that the beauty of a photo does not guarantee its popularity on social media. The idea of addressing the popularity problem as classification and regression instances was introduced in [10] by San Pedro and Siersdorfer, where classification models were used to classify photos into attractive or unattractive classes, and a regression model was used to rank a photo based on its attractiveness. They combined basic visual features and tags to predict image attractiveness defined as the number of favorites. As expected, the results showed that combining tags and visual features performed better than relying on only one feature. These studies only considered part of the factors or representations in social data. A more comprehensive study is needed.

Khosla et al. proposed more comprehensive work in image popularity prediction [12]. They built a regression model to predict image popularity based on the number of views normalized by time. They investigated the effectiveness of low level visual features, such as texture and Gist, and as well as that of deep learning features. In addition to the content features, they leveraged the social clues related to the users and images; for instance, contacts, groups, tags and the lengths of titles and descriptions. Together, visual and social features led to a better performance than individual features. Following a similar approach as [12], in [14], Gelli et al. utilized visual sentiment features and high level features (objects). They also used social features and textual features to recognize named entities from the text attached to the images and referred to them as tag type and tag domain.

Yamasaki et al., in [9], proposed to predict image popularity scores by utilizing tag information. They computed the tag’s importance by combining the tag frequency and weight learned from support vector regression. This method obtained better results than predicting popularity based on tag frequency scores. Their approach is cost effective but fails to predict the popularity score if there is no tag information. Learning the rank of an image, in terms of popularity based on visual features, was introduced in [13] by Cappallo et al. They used deep learning features to discover latent scenes that affect the popularity of images. Another approach was proposed by Wu et al. [25], who predicted image popularity using a matrix factorization technique. They utilized the temporal information of the interaction between users and images.

Prior works considered various types of features related to image content (content based) and/or social features. These features helped predict a social image’s popularity by addressing the problem as a learning problem with different paradigms. There is no unified definition of image popularity or of evaluation metrics due to the difference in the way the problem is formulated by different groups. In this work, we first analyze a real-world dataset collected from Flickr to understand human social interactions, and subsequently identify the social clues that are correlated with image popularity and visibility within the system. Based on our data analysis, we propose a multi-modal image popularity prediction algorithm which utilizes multi-level visual features, social context, and textual information.

## 3. Data Collection and Analysis

Data collection procedures are described in Section 3.1, and data analysis is discussed in Section 3.2.

### 3.1. Data Collection

To collect images, we utilized groups from Flickr, where images are organized by theme and are uploaded by various registered users. One reason to choose groups as a medium to collect images is the differences in image quality and social clues. We selected 31 topics covering a wide range of visual concepts that are listed in the NUS-WIDE dataset [26] and in Flickr’s popular tags [27]. Examples of these topics are animal, bike, bridge, cloud, food, football, lake, plane, reflection, tree, wedding, and winter. We used the selected topics as text queries to return lists of groups wherein these visual concepts are represented. For every query, we filtered the list of groups returned to ensure that the groups are public and reflect the concepts. Then, we selected 10 groups for each visual concept, for a total of 310 groups. Images belonging to these groups are downloaded, as is the social information related to these photos. The images and their social context are collected through the Flickr API. Our dataset consists of 1.5 million images uploaded by 90,532 users: we refer to this set as the original dataset. The dataset consists of images with significant variation in the number of views. The maximum number of views an image has received in the dataset is 1,603,158, while 121 images have received the minimum value of views which is equal to zero. The median number of views and the mean are 385 and 1147.96 respectively. Only 0.02% of the images have received views over 100 K, while 16% have obtained less than or equal to 100 views in our dataset. In addition, the mean number of comments and favorite are 12.7 and 16.2 respectively. Similar to the views, the minimum number of comments and favorite an image has received in our dataset is zero. The maximum number of comments and favorite an image obtained were 10,541 and 14,694 respectively. 77% of the images in our dataset received less than 10 comments and, following the same pattern, 72% of the images had number of favorite less than 10. Only 0.07% of the total number of images has more than 1000 comments. Similarly, 0.06 % of the images obtain more than 1000 favorite.

### 3.2. Social Interaction Analysis

**Figure 2:**
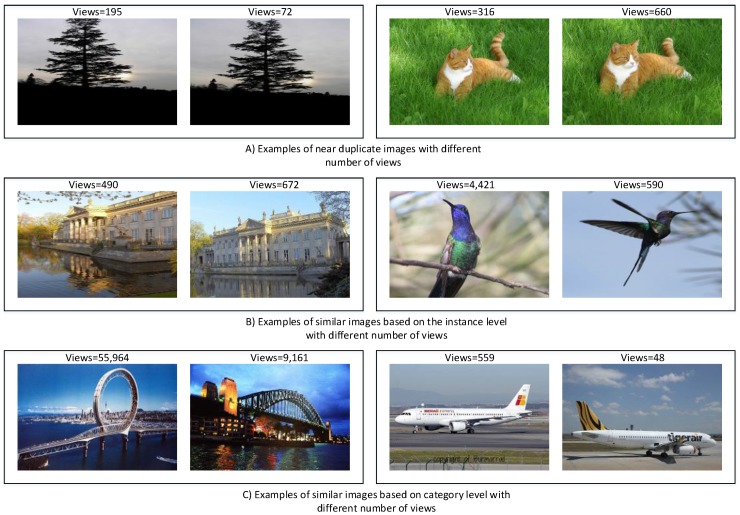
Examples of visually similar images. Despite the similarity, they receive a significant different number of views.

Social interactions in online social media are classified into explicit or implicit interactions. Explicit interactions require time and effort from the registered users and are denoted by the number of comments and favorites on the Flickr platform. Implicit interactions are represented by the number of views; however, this social metric is not necessarily a reflection of Flickr members’ interest. Hence, the gap between explicit and implicit interactions is significant, yet they have a strong positive relationship. This is a valid observation in our dataset, and the correlation between the number of views and the number of comments equals 0.48. From our dataset, we also observe that images which include similar instances or objects may not acquire a similar level of social interactions. In Figure 2, we illustrate three examples of visually similar images which, despite the similarity, received different numbers of views: (A) near duplicate images; (B) images share the same instances; and (C) images belong to the same category. This highlights the fact that visibility on social media is not solely dependent on visual content. The following questions arise: If visual content is not sufficient to increase social interaction, what are other factors that influence interactions on social media? How can we leverage these factors that increase image visibility and subsequently boost social interactions? Thus, understanding online social behavior is a fundamental step in the prediction process. In order to address these questions, we analyze the interactions between users and images, considering how users browse the social media sites. This leads to three main points that need to be investigated: Do users find images only by browsing the images uploaded by their contacts or do they find interesting images through survey groups that cluster photographs based on themes? Finally, how does searching for images using keywords affect the visibility of images and the number of social interactions? To find the answers, we construct two samples of images from our original dataset. The first set represents photos with a high number of views; we refer to this set as the representative dataset. The other set of photos contains randomly selected images with a varying number of views to represent the distribution of image popularity; this set is denoted as the random dataset. The descriptions of the two datasets are provided below:

- Representative dataset: Images in our original dataset are ranked based on the number of views and then, the Top-1000 photographs are selected to represent a set of the popular images in our dataset.
- Random dataset: We randomly select 50,000 images from our original dataset that are divided into 10 sets where each set consists of 5000 images. The selected images have varying numbers of views. The most popular image among the 10 sets of images has 1,243,643 views, whereas some images have zero views. In Table 1, we provide a brief description of our datasets. For both datasets, we collect the social information of the owners and the images in addition to the metadata of the photos.

To begin, because of the limited access to the viewers of images on Flickr, we analyze the source of comments and favorites that images received in both the representative and random datasets. Table 2 illustrates the percentage of interactions that images obtained from two sources: groups and user contacts. In both datasets, photos obtained the majority of their interactions from group members. In the representative set, images received 53% of the comments from the groups and only 13% from their owners’ contacts. Following the same pattern, the randomly selected images acquired 70% of comments and 75% of favorites from the groups that they joined. We observe that contacts have fewer interactions with images than do group members; however, this can be justified because we only consider contacts that the owners of images are following, as Flickr is a unidirectional network. The information of the reverse contacts, i.e., who follows a user, is not directly available through the Flickr API. In addition, overlap between contacts and group members is typical in social media, yet the overlap is minimal in our case because we consider the users’ contacts and not their followers. Nonetheless, a small percentage of interactions are from other users who are not in the groups or the contact lists. The results highlight that the majority of interactions are from groups, which signals the importance of sharing images with groups. Notably, groups vary in popularity and activity level, which affect the number of social interactions; thus, we investigate the popularity power of groups and users on image popularity. Furthermore, we explore the impact of assigning tags and text to images to make the image more accessible and visible in search results. This analysis is provided in Section 3.2.1 and Section 3.2.2.

#### 3.2.1. Social Context and Social Interaction

The statistics presented in the last section provide some insight into social interactions; however, we need to understand the impact of groups and users popularity on the social interactions. A user’s popularity and activity level can be inferred from several social factors. We consider the number of contacts and the mean count of photo views as indicators of a user’s popularity, and the number of joined groups and uploaded images as the activity level. For groups, the numbers of members and shared images represent the group’s activity level. In addition, for groups, through analyzing the data, we discovered that an image can be shared with groups that are not in the owner’s list. Some of these groups are private groups and accept images by invitation only, so it is not necessary for the image’s owner to join the group. Thus, we divide the groups into image groups where the image is shared but the owner of the image is not a member, and user groups to which the user is subscribed. We compute the strength and the direction of the relationship between both types of social interactions and social factors by calculating Spearman’s correlation coefficient [28]. The range of the correlation coefficient is from −1 to +1, where a correlation ratio close to 1 or −1 indicates a positive or negative relationship, respectively. Correlation values close to zero show a random relationship. To compute Spearman’s correlation, we rank the same set of images based on different criteria: social interactions and social factors that are related to the images or to the owners. For example, we rank the images based on the number of views, number of tags assigned to the images, or number of groups the user has joined where this ranking is done independently. The value of the Spearman’s correlation is computed using Equation (1), where *n* is the number of images in the set and $d_{i}$ is the square value of the difference between the rankings of the images.

(1) $$\rho =1-\frac{6\sum d_{i}^{2}}{n(n^{2}-n)}$$

The results obtained using the random dataset are listed in Table 3. The reported results are the average calculated over the 10 sets. The table shows a strong correlation between the popularity of the user and the number of social interactions. The mean number of views of a user’s photos is strongly correlated with the number of implicit and explicit interactions (0.58 and 0.699, respectively) of a specific image. Moreover, the size of a user’s network is represented by the number of contacts, and has a strong positive relationship with the number of views “implicit interaction” (0.44). The same result also applies to comments and favorites “explicit interaction”. In addition to user context, the number of groups in which the image is shared is highly correlated with the number of social interactions. The correlation ranges from 0.524 for comments and favorites to 0.479 for views. The correlation between the number of members in image groups and social interactions is higher than with the number of members of the users’ groups. In contrast, there is no relationship between the user’s level of activity, or number of uploaded photos, and the number of social interactions.

We further list the correlation results on the representative dataset in Table 4. We can see that the user’s contacts have a greater impact on the number of comments and favorites than on the number of views. The average number of views for a user’s images has a moderate correlation with the number of views of the new image, while a random relationship is found with the number of comments and favorites. Image groups positively influence the number of comments, favorites and views. On the other hand, the number of user’s uploaded images has a negative relationship with the number of comments and favorites received. From the results, we can see there are differences between social factors correlation with implicit and explicit interactions in each of the random and representative datasets. The number of images in the representative dataset is smaller than the number of images in the random set which, could impact the correlation measurement. Images in the representative dataset belong to users with large number of followers in general, a detail which influences their images’ popularity. Unfortunately, we cannot directly crawl the followers’ information from Flickr. In addition, we noticed that the owners of these popular images within our dataset are joining relatively small numbers of groups while their images are being shared with more groups where the user is not a member. In general, it is apparent that a user’s popularity and the groups to which he belongs have an influence on the image’s popularity. Contacts and groups show a higher correlation with explicit interactions than do the views; however, the popularity of a user’s images significantly impacts the number of views. This supports our hypothesis that the scope of a user’s network and their choice of groups affect the visibility and popularity of their images in social media.

#### 3.2.2. Textual Context and Social Interaction

Another important feature of Flickr is image annotation, more specifically, tags and text associated with images. This textual information is used to index the images and describe their content to increase accessibility when searching via keywords. We analyzed the correlation between the number of tags associated with an image and the number of views and explicit interactions received by the image. The results are showed in Table 3 and Table 4. In the random dataset, the number of tags has a positive correlation with both types of interactions. The correlation between the number of tags and implicit interaction is 0.402. This value decreased to 0.233 when measuring the strength of the relationship between tags and the number of explicit interaction. This is due to the fact that explicit interaction is motivated usually by social relationship rather than finding an image by search, as is the case in implicit interaction. In the representative dataset, we can see there is a negative relationship between the number of tags associated to the image and implicit interaction. This indicates that some of the popular images are receiving views based on the user popularity; However, this does not negate the fact that assigning tags to images is an important factor to increase images visibility to other users and worth more investigation. Hence, we rank the images in the random dataset based on the number of views received in order to analyze the number of tags assigned to popular and unpopular images within this sample. From Table 5, it is obvious that the gap in the number of tags assigned to popular and unpopular photos is significant. The number of tags attached to the top-100 viewed images is 2039. In contrast, the 100 least popular images have only 82 tags. The difference between the number of tags associated with the top-500 ranked images and with the 500 least popular images is 9442.

**Figure 3:**
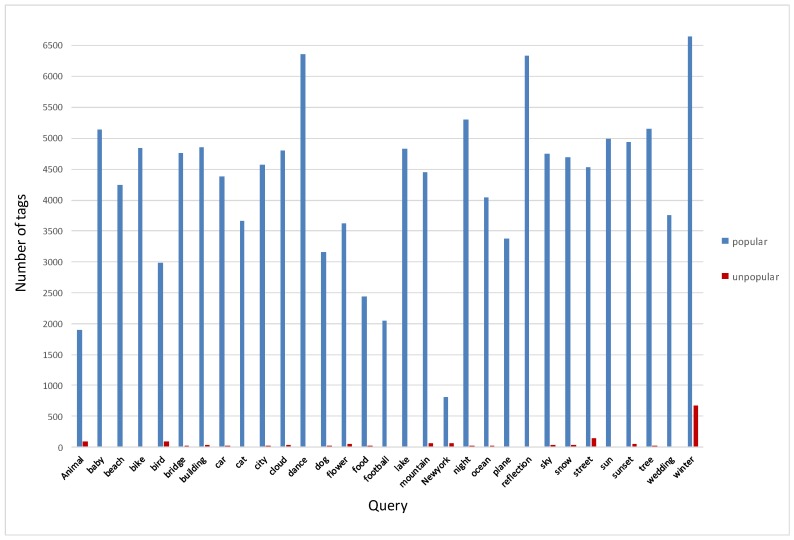
The gap between the number of tags associated with popular photos and unpopular photos.

**Figure 4:**
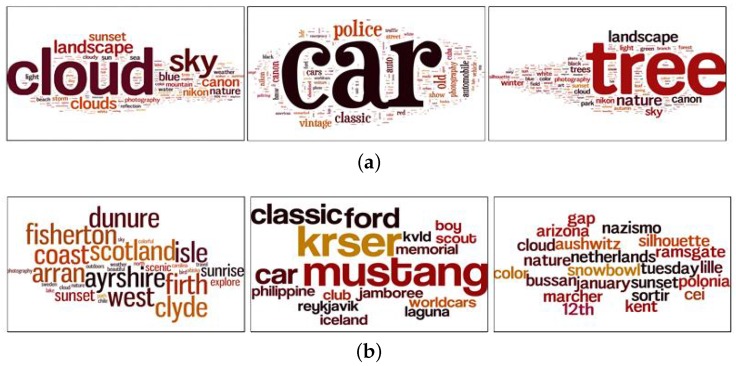
Tagclouds generated from popular and unpopular images on Flickr. (**a**) text associated with popular photos, (**b**) text associated with unpopular photos.

We went a step further and collected new images from Flickr to analyze the text assigned to these images. We searched for images using the same queries that we used to return the group images (31 different visual concepts) and returned photos based on interesting values calculated by Flickr. Through this step, we obtained two sets of images, interesting images and uninteresting images, based on Flickr’s interestingness score. Comparable to our findings using our datasets, the difference in the number of tags is significantly high. Figure 3 shows a comparison of the number of tags assigned to the 1000 most popular and most unpopular images belonging to the same visual category. Moreover, we analyzed the text used to describe popular and unpopular images and generated tag-clouds to depict the most frequently used words. Figure 4 presents the tags associated with popular and unpopular photos; we can see that popular tags are typically common words such as cloud, car, and tree. The majority of unpopular images lack titles and descriptive text and are associated with fewer tags, if any. In general, these tags are more specific, in that they describe specific attributes regarding the image visual content, such as the car’s brand. Furthermore, the text is not comprehensive, and only covers a portion of the visual content and the semantics of the images; unlike interesting images that are rich in textual information. Most of the popular images are uploaded with informative titles and descriptions. In addition, the photos are annotated with tags that describe visual content besides human semantic concepts. These tags are more descriptive and include more searchable keywords, which increases the chance that the images will appear in the search results. The same observation applied to our datasets in terms of tags and description quality.

In summary, the data analysis suggests that popularity on social photo-sharing sites is influenced by a user’s reachability and the use of the features provided by the service providers. We found out that most of the comments and favorites are from groups members in both datasets. Also, in the random dataset, we can see high correlation with both types of social interactions and the number of users’ contacts. In addition, tags attached to the images are important in finding the images on Flickr via keywords search. Hence, we can conclude that users browse images and explicitly interact based on their interests and social relationships. At the same time, we cannot neglect that users will use keywords to view images of interest. Here, we conclude that, on Flickr, the number of social interactions an image receives depends on the popularity of the user, the groups selected, and the quality of the text associated with the image. These factors collaboratively increase the popularity of images and play an important role in prediction applications on social media. In the next section, Section 4, we demonstrate how we applied our findings with a combination of content features in order to effectively predict the popularity score of social images.

## 4. Image Popularity Prediction

The popularity of social images is measured by various social signals, depending on the social interactions supported by the social media sites. For example, on Facebook, the popularity can be measured by the number of likes or comments, whereas on Twitter, it is measured by the number of re-tweets. Previous works addressed popularity prediction as a regression or classification problem. In this work, we target Flickr as the main platform for predicting a social image’s popularity, where the popularity is related to social interaction behaviors. Consequently, we define an image’s popularity based on its received number of explicit or implicit interaction. Explicit interactions are represented by the number of comments and favorites, where users explicitly express their interest in an image. In our work, we refer to these as “interactions”. Implicit social interactions are defined simply as the number of views. At this point, we formalize popularity prediction as learning to rank images on Flickr based on their popularity score in terms of the number of views or the number of comments and favorites. Because our dataset consists of images with dramatic variations in their number of views and interactions, we apply the log function. Images receive social interactions during their time online, so to normalize the effect of the time factor we divide the number of interactions an image has obtained by the number of days since it was first uploaded on Flickr. This is known as a log-normalization approach, which is proposed by [12], and defined in Equation (2).

(2) $$score_{i}=log_{2}\frac{\left(p_{i}+1\right)}{T_{i}}$$

where $p_{i}$ is the popularity measure. In the following, we consider two types of popularity measures: (i) “views” is the number of views; (ii) “interaction” is the sum of the number of comments and the number of favorite. Comments and favorite have comparable values and explicitly show the users interests thus we consider them as a measure of popularity. $T_{i}$ is the time duration in days since the uploaded date on Flickr.

**Figure 5:**
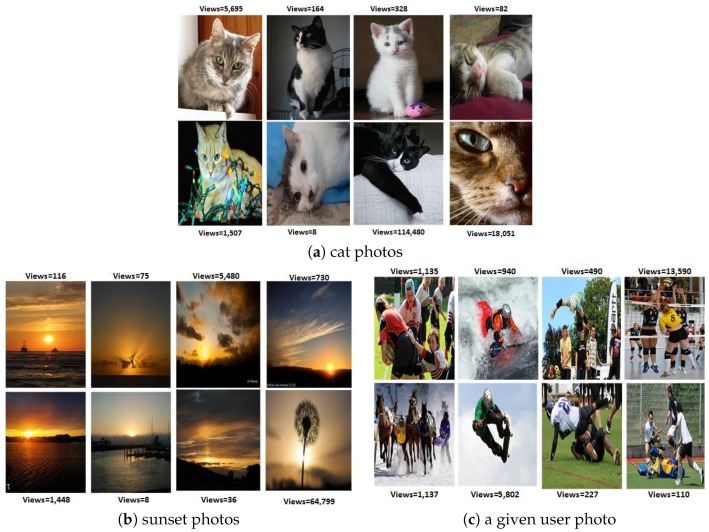
Variation in the popularity metric “views” for images with similar visual concepts and a user’s collection.

**Figure 6:**
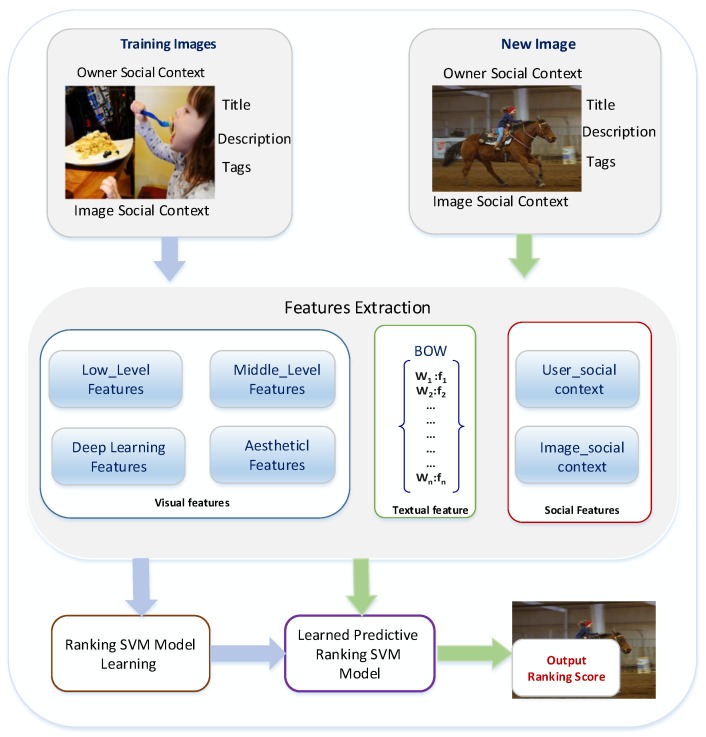
The framework for predicting popularity.

From our data analysis, we observe that the number of views varies between images within users’ collections and groups. Figure 5 illustrates an example of this inequality in image popularity scores. Thus, we are proposing a popularity prediction algorithm utilizing multi-modal features. We investigate the effect of different visual features that are designed to represent different visual aspects of images, including visual variances and visual semantics. In addition, we consider the impact of an image’s beauty, where we hypothesize that if images are similar in terms of visual content and social cues, then the beauty will play an important role on the popularity of the images. Moreover, we explore the role of contextual and textual factors in predicting an image’s popularity. In our approach, we follow the standard framework for prediction, which consists of two main components: feature extraction and model learning. This framework is depicted in Figure 6. Given a training set of images, we extract different types of features to represent the images. Then, in the model learning stage, we utilize the Ranking Support Vector Machine (Ranking SVM) [29] to be trained on our dataset, and the learned model will be used to predict image popularity ranking score for a new set of photos. In the following sections, we briefly introduce the Ranking SVM algorithm and provide the details of the features that are used in our work.

### 4.1. Ranking SVM

We consider the problem of popularity prediction as a pairwise learning to rank problem. In the pairwise technique, the ranking problem is reduced to a classification problem over a pair of images, where the objective is to learn the preference between the two images. In our experiment, we apply the l_2 regularized l_2 loss function Ranking SVM algorithm to learn the preference between a pair of images with the linear kernel implemented using the LIBLINEAR library [30].

In Ranking SVM, a set of training images with labels is given as $X=\left(x_{1},y_{1}\right),\left(x_{2},y_{2}\right),...,\left(x_{n},y_{n}\right)$, where $x_{i}$ is a *d*-dimensional feature vector of image *i* and $y_{i}$ is the popularity score of image *i*. There exists a preference order for a pair of images such that “$x_{i}$ is preferred to $x_{j}$” is denoted as $x_{i}>x_{j}$ when $y_{i}>y_{j}$. The objective of a ranking function is to return a score for each data point, an image in our case, where a global ranking over the data is generated. Thus, the ranking function *F* outputs a ranking score for images such that $F\left(x_{i}\right)>F\left(x_{j}\right)$ for $y_{i}>y_{j}$, which minimize the given loss function. *F* is assumed to be a linear ranking function:

(3) F(x)=w×x

to learn *F*, the weight vector *w* should be computed for most of the pairs such that:

(4) $$\begin{array}{c} F\left(x_{i}\right)>F\left(x_{j}\right)\Rightarrow \left(w\cdot x_{i}>w\times x_{j}\right)\\ F\left(x_{i}\right)>F\left(x_{j}\right)\Rightarrow w\left(x_{i}-x_{j}\right)>0 \end{array}$$

Now, the relationship between the pair of images $x_{i}$ and $x_{j}$ is represented by the new vector $x_{i}-x_{j}$. The relationship between any pair of images can therefore be represented by a new feature vector and new labels as follows [29,31,32,33,34]:

(5) $$x_{i}-x_{j},l=\left\{\begin{array}{cc} +1 & \;y_{i}>y_{j}\\ -1 & \;y_{j}>y_{i} \end{array}\right.$$

The problem now becomes a classification problem using SVM that can assign positive or negative label to any vector $x_{i}-x_{j}$.

### 4.2. Image Visual Content Features

To investigate the effect of an image’s content on its popularity, we consider various types of visual features that describe different perspectives of the image. We use Low level computer vision features that efficiently describe the visual appearance of the image, extracted directly from the pixel information. Although low level features perform well when describing the image, they fail to interpret the semantic of the image. Thus, we leverage middle level features that are designed to address the semantic and affective gaps. In addition to these features, we adopt Aesthetic features to represent the beauty of the image, and we extract high level features that detect the appearance of objects in the image using a deep learning technique.

#### 4.2.1. Low level Features

We consider extracting four low level features, described as follows:

- Color Histogram: We extract the RGB color channels to represent the color distribution of the image. The three color histograms are concatenated to form one feature vector of 768 dimensions [35].
- Local Binary Pattern (LBP): A famous texture descriptor widely used in computer vision applications. It works by comparing the value of each pixel with its 8 neighbors in a 3×3 neighborhood. If the value of the selected pixel is greater than its neighbors’ values, the neighboring pixels are encoded with 1; otherwise, they take on a value of 0. This results in eight binary numbers that are concatenated in a clockwise direction to calculate the corresponding decimal value of the selected pixel [36,37]. Ojala et al. [38] recognized that certain patterns are more informative than others, which resulted in the introduction of the uniform LBP. In our work, we used the uniform LBP descriptor, which resulted in 59-dimensional features.
- GIST: A popular descriptor in recognition applications and image retrieval, proposed by [39]. GIST recognizes the scenes in images based on the formation of spatial representations that summarize the description of the image scenes, such as layout and category, with few objects. We adopt the GIST descriptor, where the image is divided into a 4x4 grid, and the orientation of the pixels is computed by a Gabor filter. The use of the GIST algorithm resulted in a 320-dimensional feature vector [39,40].
- Bag_of_Visual_Word (BoVW): A widely used feature in image classification inspired by the famous Bag_of_Word feature (BoW) used in information retrieval and text mining. Due to the differences between images and discrete words in textual documents, the images are treated as patches of representative samples, where the keypoints are detected by applying the SIFT descriptor. The descriptors are then grouped into clusters, where each cluster represents a quantified visual word. Finally, the images are represented as BoVW vectors based on their vocabulary distribution [41,42]. In our work, we follow the same approach presented in [43] which consider a 2-layer spatial pyramid and max pooling strategy to generate the BoVW. This resulted in feature vectors of 1500 dimensions.

#### 4.2.2. Middle level Features

We have explored middle level features that represent different semantic concepts present in the images. Moreover, we consider features that detect the emotions and sentiments that appear in the visual content. The selected features are the following:

- Classemes: A descriptor that is built to detect objects in images. This descriptor is a combination of the outputs of classifiers trained to detect 2659 object categories. These categories consist of visual concepts, including concrete names or abstract nouns, and are suitable for a general_category image search. The category labels are selected from the Large Scale Concept Ontology for Multimedia (LSCOM), which is designed for image retrieval [44].
- Category_Level Attributes: Represents properties shared across categories that can be described using adjectives, such as long and red (in the case of hair), instead of concise object names. In our work, we extracted the attribute features based on the technique introduced in [45], which resulted in a feature of 2000 dimensions.
- SentiBank: Unlike Classemes and Attribute, SentiBank is a middle level feature designed to leverage the affective gap between low level features and the sentiments present in the visual content. SentiBank is designed based on Visual Sentiment Ontology (VSO) and can detect 1200 Adjective_Noun Pairs (ANPs), such as ’peaceful lake’ and ’smiling eyes’, that are shown in images [43].

#### 4.2.3. Aesthetic Features

In addition to semantic concept detection, we investigate the impact of the image’s beauty on its popularity. We adopt Aesthetic features that are based on psycho-visual statistics rather than those based on art principles, as proposed by Bhattacharya et al. in [46]. These features are designed for videos, where the features are extracted at the cell, frame, and shot levels. Because we apply the features to images, we only consider cell- and frame-level features. This is a 149-dimensional feature vector.

#### 4.2.4. Deep Learning Features

Deep learning techniques have gained greater popularity in image classification and object recognition due to their promising performance. In our work, we used a Convolution Neural Network (CNN) architecture that is trained to classify 1.3 M images from the ImageNet challenge into one of 1000 object categories [47]. More specifically, we used the CAFFE [48] framework of the CNN to extract the following features:

- FC7: We extract features from the last fully connected layer (FC7), which is the layer located directly before the classification layer. The output of this layer is a 4096 dimensional features vector and is considered to be a middle level feature.
- FC8: High level features that are represented by a 1000-dimensional feature vector representing the output of the classification layer, which can distinguish between 1000 objects.

### 4.3. Contextual Features

The data analysis provided in Section 3.2 shows a social image’s popularity is not only dependent on the visual content, but the contextual factors play a primary role on an image’s popularity as well. We define the contextual features as the statistical information about the images and their owners on photo-sharing social networks. Contacts and groups where the images are shared with people interested in similar content show a strong positive influence on the number of views and interaction that the images will receive. Consequently, we must consider different contextual factors that impact the image popularity. We have categorized the contextual features into owners’ features and images’ features. The owner’s contextual factors that are correlated with popularity and considered in our experiments are the number of contacts, total number of uploaded photos, and average number of views of the uploaded images. In addition, we consider the number of groups a user subscribes to, the average number of group members, and the average number of images belonging to the groups. We choose to use the number of group members and the number of images to indicate the popularity and activity level of the groups. Furthermore, we consider the contextual features of images that are provided by the images’ owners: the total number of groups, the average number of members participating in these groups, and the average number of photos shared with the groups. The decision to select image groups as a contextual feature was made based on our observations from the data analysis, where we saw that images could be shared with groups that were not in the owner’s list. In addition, we include the number of tags associated with the image as a context feature of the image. We decided to exploit the number of tags assigned with images because intuition led us to believe that an image with more tags will appear more often in search results. We combined all the social features after applying l_2 normalization to generate one feature vector with 10 dimensions.

### 4.4. Textual Features

Images are not always given descriptive tags, and the quality of the tags cannot be neglected [49]. This is clearly demonstrated in our data analysis. Thus, we explore the effect of text attached to the image on its popularity. We have used the basic textual feature Bag_of_Word (BOW), which is heavily used in text mining due to its simplicity and good performance. Each image is represented as a feature vector of length *n* where each element is corresponding to a term in a pre-defined vocabulary. This feature vector can be represented as binary or frequency vector. To generate the vocabulary, we consider two schema: Term Frequency (TF) to select the most frequent terms appear in images tags, title, and description, and Term frequency-Inverse Document Frequency (TF-IDF) that reduce the weight assigned to more frequent terms. Before selecting the vocabulary, we applied essential natural language preprocessing steps such as removing the stop words and URLs. Also, we applied the Word-Net Lemmatizer provided in the NLTK [50]. The vocabulary used to generate the BOW is of size 1000 terms. Thus, we have a feature vector with 1000 dimensions. This setting of the vocabulary size is the best choice for our problem based on experimental results.

### 4.5. Fusion Techniques

In previous sections, we presented the features that have been selected to individually predict an image’s popularity. In this section, we provide the details of combining multi-modal features, where we aim to boost the performance of the prediction algorithm using fusion techniques. We use the following methods:

- Early_fusion: Also known as feature_level fusion. We have normalized the features vectors using L2_normalization and then concatenated the normalized features to generate a single feature vector. Then, the learning stage is performed on the multi_representation vector.
- Late_fusion: Integrates the normalized output scores obtained from the learned models based on individual features. In our work, we applied the average late fusion method. This type of fusion is also known as decision_level fusion [51,52].
- Borda Count method: A rank-based technique that is widely used in meta-search and merging ranked lists. This method is based on a voting process, where each voter ranks a set of *n* candidates based on his/her preference. For each voter, the top-ranked candidates receive *n* points, the second ranked candidate receives n−1 points, etc. The total number of points for each candidate is calculated from all voters and then used to rank the candidates [53,54]. Because the outputs of our learned models are based on scores, we transfer them to a rank according to their scores obtained from each model. Then, we can apply the Borda Count method to merge the ranked results.
- Weighted Fusion method: A Score-based data fusion approach that is used in information retrieval systems. This method calculates the total score of a document *x* based on the weight $w_{i}$ that is assigned to each of the *k* retrieval system and the score of *x* obtained by the system [55]. The calculation of the weighted fusion method is given by Equation (6).
  (6) $$S\left(x\right)=\sum_{i=1}^{k}w_{i}\times s_{i}\left(x\right)$$
  where the weight $w_{i}$ is calculated as $w_{i}=\frac{c_{i}}{\sum_{i=1}^{k}c_{i}}$, in which $c_{i}$ is the Spearman’s correlation ratio of system *i*’s performance.

To combine the individual features to improve the performance of the prediction algorithm, we perform the sequential strategy to select the features. In this approach, we rank the features based on the achieved performance correlation. Then, starting from the best feature, we add an additional feature to be integrated based on its rank. If the added feature boosts the performance, we add the next feature until there is no improvement in the performance; when such is the case, we discard the feature that did not generate a further improvement. We select this approach of features combination since we have many different features and it has been used successfully in [56,57,58].

## 5. Experiments

In this section, we present the data settings used in our experiments and the results of the discussed algorithms. In addition, we show the effect of combining the multi-modal features. For performance comparison, we compare our proposed approach with different learning methods.

### 5.1. Experimental Setup and Evaluation Criteria

We have conducted experiments in two scenarios: The first scenario depicts the case that occurred in search results where, most often, returned images belong to different users. We denoted the dataset that represents this setting as one_per_user. In the second scenario, we predict the image’s popularity within a given user’s collection of photos. These scenarios are typical on social media sites and similar to the settings of previous work [12]. The two data sets are sampled from our original dataset, which consists of 1.5 million images. The settings of our datasets are described as follows:

- one_per_user: In this setting, we randomly select one image for each user in our dataset, resulting in a total of 89,663 images. This dataset is divided into 30,000 images used for training our model and the remaining 59,663 images for testing. This setting is suitable for investigating how the differences in visual, social, and textual factors will impact popularity.
- personal_collection: This is a personalized setting. We select the Top-80 users based on the number of images that they contributed to our dataset. The 80 users have in total 155,968 images; for each user, 60% of the photos are used for training, and 40% are used for testing. Collecting all the social context and textual information for all the users and images will grow the data size and due to the limitation in computational resources, we select the top 80 users. We train and evaluate the Ranking SVM algorithm for each user independently. The reported results are the average of the 80 users. In this setting, we follow [12] by discarding the user’s contextual factors such as contacts, mean views, and total number of uploaded photos since they are identical for all the images that belong to a specific user. We only consider the contextual features of the images that can be different among images even when they belong to the same user such as: an image groups and tags. This is because each model is trained for a specific user to predict the popularity of images belong to this specific user.

To evaluate the performance of the prediction algorithm, we use Spearman’s coefficient to compute the ranking correlation between the predicted ranking scores of the images and the ground truth scores obtained from Flickr. Spearman’s correlation equation was described in Section 3.2, Equation (1).

### 5.2. Effect of Different Features

In this section, we present empirical results that demonstrate the effect of using different modalities on the prediction of an image’s number of views and interactions, which, in turn, indicate the social image’s popularity. In Section 5.2.1, we report the results of using only image content features. A discussion and analysis of the performance of contextual and textual features in the prediction of an image’s popularity are provided in Section 5.2.2. Furthermore, in Section 5.2.3, we investigate the effect of fusing different features on the algorithm’s performance, where we observed some improvements.

#### 5.2.1. Results of Visual Feature

The results are illustrated in Table 6 for the one_per_user and personalized_collection settings. For the one_per_user dataset, low level and Aesthetic features have achieved lower performance than the other features. For low level features, BoVW which is known for its performance in object recognition, has provided the best results in predicting the number of views and interactions. LBP a powerful feature that is used in face detection, achieves a slightly lower rank correlation than BoVW on predicting views and interactions. Color, and Gist features have not help in predicting image popularity. When comparing middle level and deep learning features, both SentiBank and FC7 provide better results than Classemes and Attribute. This highlights the importance of emotional concepts that appear in the image visual content in predicting social image popularity. The difference in the detected visual concepts among middle level features result in the differences in the prediction algorithm performance. Classemes and Attribute detect concrete and abstract visual concepts respectively while SentiBank is designed for detecting sentiment in visual content. We observed that the high level feature FC8 is better at predicting the number of interactions than the number of views. In this setting, Aesthetic feature has achieved lower performance than middle and deep learning features. Overall, the middle level and deep learning features outperform low level features.

For the personal_collection setting, LBP has exhibited the best performance in the low level feature category. Specifically, the rank correlation is 0.35 when predicting the number of views, and the correlation is decreased to 0.27 when predicting interactions. Gist feature performance is better than BoVW on this dataset. We have collected the images using text queries thus images belong to a specific user tend to represent similar scene categories. Accordingly, the global Gist descriptor which is designed for scene detection can represent the images more accurately. However, for one_per_user setting, the images have very various visual appearance which affect the Gist performance hence BoVW has obtained better results. For middle level and deep learning features, FC7 performs the best in predicting views and interaction signals, with correlations of 0.42 and 0.30, respectively. Aside from FC7, SentiBank outperforms other middle level and high level features. In the one_per_user dataset, we can see that Aesthetic feature has exhibited a weaker performance than others. This is because the possibility of viewing one image, which is in large pool of images on the Internet, is dictated by many factors, such as the tagged words or the influence of the owner popularity. On the other hand, when comparing the images that belong to one user, beautiful images tend to receive more attention. Thus Aesthetic feature performs better than others when predicting the popularity of images in the personal_collection dataset. In contrast to the one_per_user setting, it is not only middle level and deep learning features that achieve better results; LBP and Aesthetic features show comparable performances to semantic and object features. FC8 performance is worse than others in the personalized setting. This could be related to that images belong to a specific user usually present similar visual objects. Also, there could be object categories that are not in the FC8 classification, but appear in the images. Moreover, in this setting, implicit interactions are more predictable than explicit interactions.

Despite the differences between the set of images, FC7, SentiBank, and LBP are effectual in predicting image popularity. We can see from the results that there is a variation in the performance of some features, such as Gist, BoVW, and Aesthetic, depending on the set of images. This highlights the importance of using different visual features that represent the images at different levels when predicting the image popularity on Flickr.

#### 5.2.2. Results of Contextual and Textual Features

The experimental results of contextual and textual features are shown in Table 7. When comparing the results in Table 7 to Table 6, we observe a significant improvement of the prediction algorithm using contextual and textual features over visual features. These results show conclusive evidence that contextual and textual features are effective in predicting images popularity. For the one_per_user setting, the contextual and textual features achieve a better performance when predicting the number of interactions compared to the number of views. Contextual features have a correlation of 0.55, and the textual performance provide a 0.36 rank correlation. When predicting the popularity based on the views, the algorithm performance has decreased to 0.42 and 0.35 using contextual and textual features, respectively. The ability to predict interactions more effectively than views is due to the fact that commenting on images and marking them as favorites are highly dependent on contextual factors such as an owner’s contacts and group membership. From the result, we can see that TF and TF-IDF strategies have provided similar results. This is due to that images in this setting are collected from different users and represent various visual topics. Thus, the words used to describe the images are varied depending on the images’ visual appearance and the users’ preferences.

In the scenario of personal_collection, the performance of textual features, TF and TF-IDF, surpass that of contextual factors in predicting both the number of views and interactions. TF strategy achieves a 0.75 rank correlation in predicting views and 0.52 for interactions. The performance of TF strategy is better than TF-IDF which is opposed the expectation in text classification. This can be explained by that IDF weighting schema assigned lower weight to words that appear more frequent in the text attached to the images which represent the image visual content and semantic. Assigning large weight to rarely appeared terms which do not reflect the images topic, such as “week”, “try” and “staying” are not helpful in predicting images’ popularity. While more frequent words, such as “tree”, “sun” and “swimming” are important for representing the image visual appearance and to predict its popularity. When comparing the performance of contextual features to textual feature (TF strategy), the performance of the algorithm has dropped to 0.37 and 0.31, respectively. In this setting, the decrease in the performance of contextual features is a consequence of utilizing only the image context, more specifically, the image’s group information.

#### 5.2.3. Results of the Fusion Techniques

In previous sections, we presented the ranking performance results by exploring individual features. In this section, we will provide the results of combining multi_modality features to boost the algorithm’s performance. We examine the impact of fusing visual features that represent different aspects of an image’s content on the prediction performance, denoted as Visual_Fusion. In addition, we combine visual, contextual, and textual features to evaluate the prediction algorithm’s performance. We refer to this as combination as All_Feature_Fusion. The results of the various fusion methods are illustrated in Table 8.

For the one_per_user dataset, the fusion of Visual_features results in an improvement of the performance when compared to using individual visual features. The weighted fusion method and late_fusion have achieved slightly better performance than other fusion methods; therein, obtaining a 0.35 rank correlation when predicting the number of views. In predicting the number of interactions, all the fusion methods achieved similar performance (0.35 rank correlation). When combining visual, contextual, and textual features, the early fusion method exhibits the best performance in predicting the number of interactions obtaining a 0.62 rank correlation. Weighted fusion method has showed a rank correlation of 0.55 when predicting the number of views which is the best among other fusion methods. In the personal_collection, fusing visual features leads to an improvement in the algorithm performance. The Borda count method obtains slightly higher performance than other fusion methods. However, the early fusion of all features as well as the Borda count method each fails to improve the performance over the textual feature performance when predicting the number of views. The best performance is provided by weighted fusion, which obtain a rank correlation of 0.79 and 0.57 when predicting the number of views and interactions, respectively. The results indicate that contextual, textual, and visual features lead to better performance when integrated together. Consequently, the three factors that we have considered in our study complement each other to provide a better prediction performance than simply relying on a single feature model. 

### 5.3. Results of Different Learning Methods

In this section, we evaluate the performance of different learning methods by replacing the Ranking SVM with Support Vector Regression (SVR), which is adopted in previous works [12,14].

The results of SVR model are illustrated in Table 9 for the one_per_user and personal_collection settings. In one_per_user setting, while using visual features, the best performance results are achieved by FC8, SentiBank, and LBP features. In personal_collection, in addition to SentiBank and LBP, FC7 and Aesthetic have exhibited better performance levels than other visual features. When comparing different modalities features, contextual and textual features outperform the visual features. In general, the results of SVR performance and the results of Ranking SVM which presented in Section 5.2, demonstrate that some visual features, such as SentiBank, LBP, and FC7, are consistently perform better than other features. In addition, contextual and textual features are very effective in predicting an image’s popularity. As showed in Table 10, we further examine the results of different fusion techniques using SVR method. Combining different levels of visual features successfully enhance the performance of the prediction algorithm over simply using individual features. When using Borda Count method to combine different levels of visual features in order to predict the number of views in one_per_user setting, the performance increases from achieving only 0.115 rank correlation to 0.167. Moreover, the fusion of multi-modal features is better than depending on single modal approach.

When comparing the performance of SVR model and Ranking SVM model, listed in Table 6 and Table 7, we observe that Ranking SVM exhibits better performance for both dataset settings. This improvement is due to the selection of the prediction model, and not the selection of the features. Since SVR is built to predict the exact popularity score, there is a loss of some accuracy, as opposed to Ranking SVM, which orders the images based on their popularity scores.

We also compare our proposed approach of utilizing multi-modal features with Ranking SVM against using visual and contextual features with SVR model as used in [12]. Results are reported in Table 11. Our proposed system performs much better than the baseline method. The improvement is derived from the utility of heterogeneous social sensory data and more powerful learning method.

To sum up, the experimental results show that the utilization of multi-modality features leads to better performance in predicting an image’s popularity on Flickr. While contextual and textual features exhibit better performance than visual features since the popularity on social photo-sharing sites is impacted by other factors than visual content, we cannot neglected the visual content of the image when predicting popularity. Consequently, adopting a multi-modality approach in order to predict image popularity on photo-sharing social networks is more effective than single model approach.

## 6. Conclusions

In this study, we analyzed the social interaction behavior between online users and social images. Our analysis demonstrated that features provided by social networks facilitate the visibility of social images and boost the social interactions. Sharing images with groups and annotating them with descriptive tags help expand their visibility and reach more online users. In addition, evidence shows that, most popular images have a title and description that describe the content and the semantic behind the photographs; whereas, uninteresting images neglect this aspect. Moreover, a user’s popularity influences the popularity of their images, where users with larger social networks and popular images will receive more interactions than inactive users. Notably, a user’s images are not all equal in their popularity score. Thus, we propose to predict an image’s popularity on Flickr by considering three factors: image content, user and image contextual cues and textual features. We conducted extensive experiments on real dataset to evaluate the effectiveness of each individual features as well as their combination. The experimental results show the effectiveness of visual, contextual, and textual features when predicting an image’s popularity. Furthermore, combining multi-modal features boosts the prediction algorithm’s performance. Consequently, our proposed method of utilizing multi-modal approach to predict an image popularity on photo-sharing social networks is more effective than single modal approach.

In our study and as an initial step, we considered the internal factors that can affect the social popularity of images. Our work can be extended by considering the external factors that influence the popularity of media content such as real-world events. In addition, we could customize the popularity prediction to specific geographical regions and predict popular images based on cultural factors. It would be interesting to see how different aspects would affect the popularity of images based on geographical location and cultural background. Understanding these factors that impact image popularity and mining people’s opinions on these popular photos will help us in providing better services to enhance users’ living environments, especially if they are related to real-world issues. Possible future work could include topic popularity and product sales that can be predicted using early uploaded images which have an impact on economic and marketing sectors.

## Figures and Tables

**Table 1:** Dataset descriptions.

Dataset	Description
Original Dataset	consists of images collected from Flickr groups
Representative Dataset	subset constructed from the original dataset including the ranked Top_1000 based on the number of views
Random Dataset	subset of the original dataset consists of 10 sets each consists of 5000 images randomly selected with different number of views

**Table 2:** Percentage of explicit social interactions with images from groups and user contacts.

Dataset	Interaction	Contacts	Groups
Representative Images	Comment	13.09%	53.1%
	Favorite	17.4%	49.9%
Random Images	Comment	17%	70%
	Favorite	24%	75%

**Table 3:** Average and variance of correlation between social factors and social interactions on the 10 random datasets.

		Explicit Interaction	Implicit Interaction
User context	contacts	0.479\($\pm 1.2\times 10^{-4}$)	0.445\($\pm 2.5\times 10^{-4}$)
	uploaded images	0.039\($\pm 1.7\times 10^{-4}$)	0.072\($\pm 3.7\times 10^{-4}$)
	number of groups	0.348\($\pm 5.81\times 10^{-5}$)	0.349\($\pm 9.83\times 10^{-5}$)
	groups members	0.269\($\pm 1.67\times 10^{-5}$)	0.278\($\pm 9.84\times 10^{-5}$)
	mean views	0.589\($\pm 1.2\times 10^{-4}$)	0.699\($\pm 4.46\times 10^{-5}$)
Image context	number of groups	0.608\($\pm 5.38\times 10^{-5}$)	0.587\($\pm 6.09\times 10^{-5}$)
	groups members	0.524\($\pm 8.29\times 10^{-5}$)	0.479\($\pm 2.34\times 10^{-4}$)
	number of tags	0.233\($\pm 1.8\times 10^{-4}$)	0.402\($\pm 8.39\times 10^{-5}$)

**Table 4:** Correlation between social factors and social interactions for the representative dataset.

		Explicit Interaction	Implicit Interaction
User context	contacts	0.125	0.029
	uploaded images	−0.353	0.0135
	number of groups	−0.19	0.04
	groups members	−0.163	0.075
	mean views	0.083	0.255
Image context	number of groups	0.201	0.112
	groups members	0.14	0.076
	number of tags	0.138	−0.018

**Table 5:** The gap in the number of tags between popular and unpopular images.

Image Rank	Number of Tags	Image Rank	Number of Tags
Top 100	2039	Least 100	82
Top 200	4132	Least 200	146
Top 500	9932	Least 500	490

**Table 6:** Performance of visual features on the prediction of popularity for the one_per_user and Personal_collection settings.

		One_Per_User		Personal_Collection	
**Features**		**Views**	**Interaction**	**Views**	**Interaction**
**Low Level**	BoVW	0.132	0.142	0.2389	0.1779
	Color	0.093	0.104	0.2299	0.1366
	Gist	0.113	0.093	0.2562	0.1839
	LBP	0.129	0.139	0.357	0.2669
**Middle Level**	Attribute	0.212	0.212	0.3332	0.2264
	Classemes	0.213	0.207	0.308	0.2206
	SentiBank	0.26	0.264	0.3839	0.2595
**Deep Learning**	FC7	0.242	0.276	0.4222	0.3025
	FC8	0.215	0.24	0.2142	0.1663
**Aesthetic**		0.118	0.097	0.3498	0.2569

**Table 7:** Performance of contextual and textual features in the prediction of popularity.

		One_Per_User		Personal_Collection	
**Features**		**Views**	**Interaction**	**Views**	**Interaction**
**Contextual features**		0.428	0.555	0.3736	0.311
**Textual feature**	TF	0.353	0.363	0.7589	0.529
	TF-IDF	0.35	0.36	0.446	0.44

**Table 8:** Performance of fusion techniques in the prediction of images popularity.

		One_Per_User		Personal_Collection	
**Fusion Method**	**Features**	**Views**	**Interaction**	**Views**	**Interaction**
**Early Fusion**	Visual_Features	0.3371	0.359	0.5162	0.3868
	All_features	0.5126	0.6292	0.7589	0.5299
**Late Fusion**	Visual_Features	0.346	0.3566	0.5318	0.3825
	All_feature	0.5192	0.5799	0.7839	0.5683
**Borda Count**	Visual_Features	0.3371	0.355	0.549	0.407
	All_feature	0.5126	0.569	0.758	0.537
**Weighted Fusion**	Visual_Features	0.347	0.358	0.533	0.383
	All_feature	0.559	0.601	0.794	0.575

**Table 9:** Prediction results of SVR model using our features for one_per_user and Personal_collection settings.

		One_Per_User		Personal_Collection	
**Features**		**Views**	**Interaction**	**Views**	**Interaction**
**Low_Level**	BoVW	0.029	0.036	0.211	0.143
	Color	0.01	0.018	0.249	0.071
	Gist	0.031	0.024	0.252	0.142
	LBP	0.078	0.1003	0.329	0.228
**Middle _Level**	Attribute	0.056	0.059	0.308	0.163
	Classemes	0.077	0.076	0.269	0.172
	SentiBank	0.089	0.099	0.381	0.175
**Deep Learning**	FC7	0.059	0.074	0.349	0.246
	FC8	0.115	0.137	0.207	0.103
**Aesthetic**		0.064	0.068	0.328	0.231
**Contextual features**		0.381	0.503	0.323	0.263
**Textual feature**	TF	0.184	0.215	0.709	0.492
	TF-IDF	0.177	0.202	0.629	0.416

**Table 10:** Performance of fusion techniques in the prediction of images popularity using SVR algorithm.

		One_Per_User		Personal_Collection	
**Fusion Method**	**Features**	**Views**	**Interaction**	**Views**	**Interaction**
**Early Fusion**	Visual_Features	0.141	0.187	0.446	0.326
	All_features	0.381	0.503	0.739	0.528
**Late Fusion**	Visual_Features	0.138	0.228	0.485	0.304
	All_feature	0.381	0.503	0.721	0.517
**Borda Count**	Visual_Features	0.167	0.192	0.519	0.352
	All_feature	0.381	0.503	0.709	0.492
**Weighted Fusion**	Visual_Features	0.144	0.164	0.495	0.311
	All_feature	0.384	0.504	0.732	0.529

**Table 11:** Comparison of our proposed approach to Baseline method.

		One_Per_User		Personal_Collection	
**Features**		**Views**	**Interaction**	**Views**	**Interaction**
**Proposed approach**		0.5192	0.5799	0.7839	0.5683
**Baseline [12]**		0.09	0.119	0.458	0.188

## References

[1] Rosi A., Mamei M., Zambonelli F., Dobson S., Stevenson G., Ye J. Social sensors and pervasive services: Approaches and perspectives. Proceedings of the 2011 IEEE International Conference on Pervasive Computing and Communications Workshops (PERCOM Workshops).

[2] Pilato G., Maniscalco U. Soft sensors for social sensing in cultural heritage. Proceedings of the 2015 Digital Heritage.

[3] Krishnamurthy V., Poor H.V. (2014). A Tutorial on Interactive Sensing in Social Networks. IEEE Trans. Comput. Soc. Syst..

[4] Sakaki T., Okazaki M., Matsuo Y. Earthquake shakes twitter users: Real-time event detection by social sensors. Proceedings of the 19th International Conference on World Wide Web.

[5] Sang J. (2014). User-Centric Social Multimedia Computing.

[6] Naaman M. (2012). Social multimedia: Highlighting opportunities for search and mining of multimedia data in social media applications. Multimed. Tools Appl..

[7] Nov O., Naaman M., Ye C. What drives content tagging: The case of photos on flickr. Proceedings of the SIGCHI Conference on Human Factors in Computing Systems.

[8] Abdelhaq H. (2016). Localized Events in Social Media Streams: Detection, Tracking, and Recommendation. Ph.D. Thesis.

[9] Yamasaki T., Sano S., Aizawa K. Social popularity score: Predicting numbers of views, comments, and favorites of social photos using only annotations. Proceedings of the First International Workshop on Internet-Scale Multimedia Management.

[10] San Pedro J., Siersdorfer S. Ranking and classifying attractiveness of photos in folksonomies. Proceedings of the 18th International Conference on World Wide Web.

[11] McParlane P.J., Moshfeghi Y., Jose J.M. “Nobody comes here anymore, it’s too crowded”; predicting image popularity on flickr. Proceedings of the International Conference on Multimedia Retrieval.

[12] Khosla A., Das Sarma A., Hamid R. What makes an image popular?. Proceedings of the 23rd International Conference on World Wide Web.

[13] Cappallo S., Mensink T., Snoek C.G. Latent factors of visual popularity prediction. Proceedings of the 5th ACM on International Conference on Multimedia Retrieval.

[14] Gelli F., Uricchio T., Bertini M., del Bimbo A., Chang S.F. Image popularity prediction in social media using sentiment and context features. Proceedings of the 23rd ACM International Conference on Multimedia.

[15] Van Zwol R. Flickr: Who is looking?. Proceedings of the IEEE/WIC/ACM International Conference on Web Intelligence.

[16] Cha M., Mislove A., Gummadi K.P. A measurement-driven analysis of information propagation in the flickr social network. Proceedings of the 18th International Conference on World Wide Web.

[17] Lipczak M., Trevisiol M., Jaimes A. (2013). Analyzing favorite behavior in flickr. Advances in Multimedia Modeling.

[18] Alves L.O., Maciel M., Ponciano L., Brito A. Assessing the impact of the social network on marking photos as favorites in flickr. Proceedings of the 18th Brazilian Symposium on Multimedia and the Web.

[19] Lerman K., Jones L. (2006). Social browsing on flickr. arXiv.

[20] Cha M., Mislove A., Adams B., Gummadi K.P. Characterizing social cascades in flickr. Proceedings of the First Workshop on Online Social Networks.

[21] Valafar M., Rejaie R., Willinger W. Beyond friendship graphs: A study of user interactions in flickr. Proceedings of the 2nd ACM Workshop on Online Social Networks.

[22] Szabo G., Huberman B.A. (2010). Predicting the Popularity of Online Content. Commun. ACM.

[23] van Zwol R., Rae A., Garcia Pueyo L. Prediction of favourite photos using social, visual, and textual signals. Proceedings of the 18th ACM International Conference on Multimedia.

[24] Hsieh L.C., Hsu W.H., Wang H.C. Investigating and predicting social and visual image interestingness on social media by crowdsourcing. Proceedings of the 2014 IEEE International Conference on Acoustics, Speech and Signal Processing (ICASSP).

[25] Wu B., Mei T., Cheng W.H., Zhang Y. Unfolding temporal dynamics: Predicting social media popularity using multi-scale temporal decomposition. Proceedings of the Association for the Advancement of Artificial Intelligence.

[26] Chua T.S., Tang J., Hong R., Li H., Luo Z., Zheng Y. NUS-WIDE: A real-world web image database from national university of singapore. Proceedings of the ACM International Conference on Image and Video Retrieval.

[27] All time most popular tags on Flickr. https://www.flickr.com/photos/tags.

[28] Spearman C. (1904). The Proof and Measurement of Association between Two Things. Am. J. Psychol..

[29] Joachims T. Optimizing search engines using clickthrough data. Proceedings of the Eighth ACM SIGKDD International Conference on Knowledge Discovery and Data Mining.

[30] Chang C.C., Lin C.J. (2011). LIBSVM: A library for support vector machines. ACM Trans. Intell. Syst. Technol..

[31] Herbrich R., Graepel T., Obermayer K. (2000). Large Margin Rank Boundaries for Ordinal Regression. Advance in Large Margin Classifiers.

[32] Cao Y., Xu J., Liu T.Y., Li H., Huang Y., Hon H.W. Adapting ranking SVM to document retrieval. Proceedings of the 29th Annual International ACM SIGIR Conference on Research and Development in Information Retrieval.

[33] Hang L. (2011). A short introduction to learning to rank. IEICE Trans. Inf. Syst..

[34] Yu H., Kim S. (2012). SVM Tutorial—Classification, Regression and Ranking. Handbook of Natural Computing.

[35] Shapiro L.G., Stockman G. (2001). Computer Vision.

[36] Heikkila M., Pietikainen M. (2006). A texture-based method for modeling the background and detecting moving objects. IEEE Trans. Pattern Anal. Mach. Intell..

[37] Huang D., Shan C., Ardabilian M., Wang Y., Chen L. (2011). Local Binary Patterns and Its Application to Facial Image Analysis: A Survey. IEEE Trans. Syst. Man Cybern C.

[38] Ojala T., Pietikainen M., Maenpaa T. (2002). Multiresolution gray-scale and rotation invariant texture classification with local binary patterns. IEEE Trans. Pattern Anal. Mach. Intell..

[39] Oliva A., Torralba A. (2001). Modeling the Shape of the Scene: A Holistic Representation of the Spatial Envelope. Int. J. Comput. Vis..

[40] Douze M., Jégou H., Sandhawalia H., Amsaleg L., Schmid C. Evaluation of GIST descriptors for web-scale image search. Proceedings of the ACM International Conference on Image and Video Retrieval.

[41] Nowak E., Jurie F., Triggs B. (2006). Sampling strategies for bag-of-features image classification. Computer Vision–ECCV 2006.

[42] Bruni E., Tran N.K., Baroni M. (2014). Multimodal Distributional Semantics. J. Artif. Int. Res..

[43] Borth D., Ji R., Chen T., Breuel T., Chang S.F. Large-scale visual sentiment ontology and detectors using adjective noun pairs. Proceedings of the 21st ACM International Conference on Multimedia.

[44] Torresani L., Szummer M., Fitzgibbon A. (2010). Efficient Object Category Recognition using Classemes. European Conference on Computer Vision (ECCV).

[45] Yu F., Cao L., Feris R., Smith J., Chang S.F. Designing category-level attributes for discriminative visual recognition. Proceedings of the IEEE Conference on Computer Vision and Pattern Recognition.

[46] Bhattacharya S., Nojavanasghari B., Chen T., Liu D., Chang S.F., Shah M. Towards a comprehensive computational model foraesthetic assessment of videos. Proceedings of the 21st ACM international conference on Multimedia.

[47] Krizhevsky A., Sutskever I., Hinton G.E., Pereira F., Burges C.J.C., Bottou L., Weinberger K.Q. (2012). ImageNet Classification with Deep Convolutional Neural Networks. Advances in Neural Information Processing Systems 25.

[48] Jia Y., Shelhamer E., Donahue J., Karayev S., Long J., Girshick R., Guadarrama S., Darrell T. (2014). Caffe: Convolutional Architecture for Fast Feature Embedding. arXiv.

[49] Kennedy L.S., Chang S.F., Kozintsev I.V. To search or to label?: Predicting the performance of search-based automatic image classifiers. Proceedings of the 8th ACM International Workshop on Multimedia Information Retrieval.

[50] NLTK package. http://www.nltk.org/.

[51] Snoek C.G.M., Worring M., Smeulders A.W.M. Early versus late fusion in semantic video analysis. Proceedings of the 13th Annual ACM International Conference on Multimedia.

[52] Atrey P.K., Hossain M.A., El Saddik A., Kankanhalli M.S. (2010). Multimodal fusion for multimedia analysis: A survey. Multimed. Syst..

[53] Aslam J.A., Montague M. Models for metasearch. Proceedings of the 24th Annual International ACM SIGIR Conference on Research and Development in Information Retrieval.

[54] Renda M.E., Straccia U. Web metasearch: Rank vs. score based rank aggregation methods. Proceedings of the 2003 ACM Symposium on Applied Computing.

[55] Vogt C.C., Cottrell G.W. (1999). Fusion Via a Linear Combination of Scores. Inf. Retr..

[56] Jiang Y.G., Xu B., Xue X. Predicting Emotions in User-Generated Videos. Proceedings of the AAAI Conference on Artificial Intelligence.

[57] Jiang Y.G., Wang Y., Feng R., Xue X., Zheng Y., Yang H. Understanding and Predicting Interestingness of Videos. Proceedings of the AAAI Conference on Artificial Intelligence.

[58] Tu J., Wu Z., Dai Q., Jiang Y.G., Xue X. Challenge Huawei challenge: Fusing multimodal features with deep neural networks for Mobile Video Annotation. Proceedings of the 2014 IEEE International Conference on Multimedia and Expo Workshops (ICMEW).

